# Calcificação Caseosa do Anel Mitral: Diagnóstico Pós-Transplante Cardíaco

**DOI:** 10.36660/abc.20210906

**Published:** 2022-11-23

**Authors:** Bruno Jordão Chaves, Matheus Bitencourt Duarte, Luiz Guilherme Passaglia, Claudio Gelape, Paulo Hernane Rabelo Azevedo, Geraldo Brasileiro

**Affiliations:** 1 Universidade Federal de Minas Gerais Faculdade de Medicina Belo Horizonte MG Brasil Universidade Federal de Minas Gerais - Faculdade de Medicina, Belo Horizonte, MG – Brasil

**Keywords:** Insuficiência da Valva Mitral, Calcinose, Neoplasias Cardíacas/cirurgia, Transplante de Coração, Cardiomiopatias, Diagnóstico por Imagem/métodos

## Introdução

A calcificação caseosa do anel mitral é uma lesão cardíaca não neoplásica, considerada uma variante da calcificação do anel mitral que deve ser suspeitada quando massas cardíacas são detectadas por ecocardiograma, radiografia de tórax ou outros estudos radiológicos.^1–3^ A maioria dos paciente é assintomática, mas já foram descritos sinais e sintomas de insuficiência mitral, embolização sistêmica e bloqueios atrioventriculares.^3^ O diagnóstico é confirmado pelo exame anatomopatológico e o prognóstico é bom. Aqui, apresentamos um caso de calcificação caseosa do anel mitral diagnosticado após a análise patológica do coração explantado.

## Relato de caso

**Figura 1:**
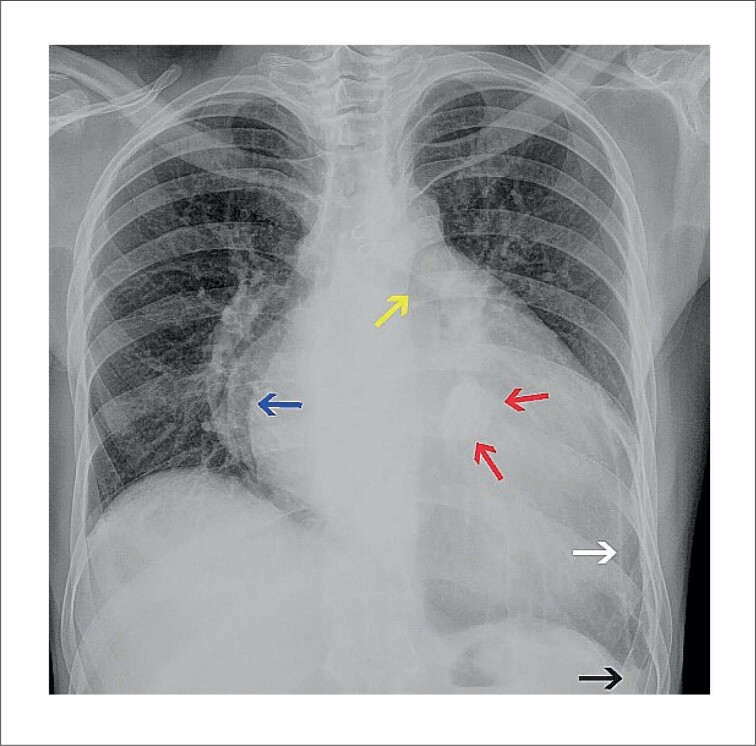
Radiografia de tórax – vista pósteroanterior. Coração aumentado. Junto à valva mitral, há uma lesão oval hiperdensa (setas vermelhas) medindo cerca de 3,5cm x 2,0cm. O sinal de dupla densidade (seta azul) e a elevação do brônquio esquerdo (seta amarela) indicam aumento do átrio esquerdo. O recesso costodiafragmático esquerdo está obliterado (seta preta), e uma linha calcificada é observada no hemitórax esquerdo, aparentemente ao longo da pleura (seta branca).

Um homem de 35 anos, com diagnóstico de insuficiência cardíaca (classe III da New York Heart Association) secundária a cardiopatia reumática foi admitido em um hospital universitário público terciário para transplante cardíaco. Ele vinha tendo internações recorrentes por insuficiência cardíaca crônica. Ao exame físico, apresentava edema de membros inferiores, deslocamento lateral do batimento apical, ritmo cardíaco irregular, presença de terceira bulha e sopro sistólico e mesodiastólico precoce no ápice do coração. A radiografia de tórax revelou lesão hiperdensa localizada no coração junto à válvula mitral (Figuras 1 e 2). O ecocardiograma transtorácico realizado três anos antes do transplante cardíaco revelou aumento dos átrios direito e esquerdo, bioprótese mitral normal e insuficiência valvar aórtica. O ecocardiograma transtorácico realizado dois meses antes do transplante mostrou aumento de átrios e ventrículos, insuficiência valvar mitral bioprotética moderada e insuficiência e estenose valvar aórtica moderada (dados não mostrados). Não foram observadas lesões calcificadas ao redor do anel valvar mitral em nenhum dos dois ecocardiogramas.

**Figura 2:**
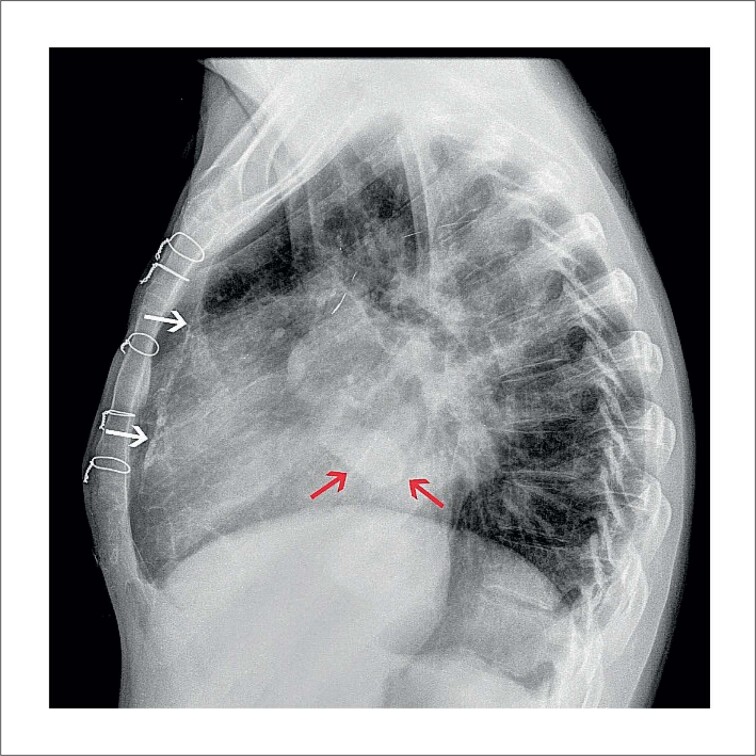
Radiografia de tórax – vista lateral. A lesão cardíaca hiperdensa (setas vermelhas) e a linha pleural calcificada (setas brancas) são visíveis.

Aos 15 anos, o paciente passou por uma troca valvar mitral por bioprótese para tratamento da doença valvar mitral reumática, necessitando de duas reoperações subsequentes (aos 21 e 23 anos). Nos últimos três anos, apesar da terapia médica otimizada, sua função cardíaca se deteriorou continuamente. Não havia outras comorbidades em sua história médica pregressa.

Os achados laboratoriais mostraram redução da função renal (creatinina sérica de 3,0 mg/dL e taxa de filtração glomerular estimada de 25,7 mL/min/1,73m²). O eletrocardiograma revelou fibrilação atrial.

A principal hipótese diagnóstica foi insuficiência cardíaca avançada causada pela disfunção valvar da bioprótese mitral. Para uma massa cardíaca visualizada na radiografia, algumas possibilidades foram consideradas: calcificação do anel mitral, mixoma calcificado, outras neoplasias ou pseudoneoplasias cardíacas, abscesso cardíaco, tuberculose, tumor amorfo calcificado ou vegetações valvares calcificadas. O diagnóstico preciso é obviamente essencial para o tratamento adequado.

### Manejo e diagnóstico

Dada a insuficiência cardíaca progressiva, o paciente foi submetido a transplante cardíaco. Durante os primeiros dias de pós-operatório do paciente, instabilidade hemodinâmica grave e insuficiência cardíaca aguda sugeriram rejeição primária do enxerto. Apesar da intervenção farmacológica para disfunção primária do enxerto, o paciente veio a óbito três dias após o transplante cardíaco.

**Figura 3:**
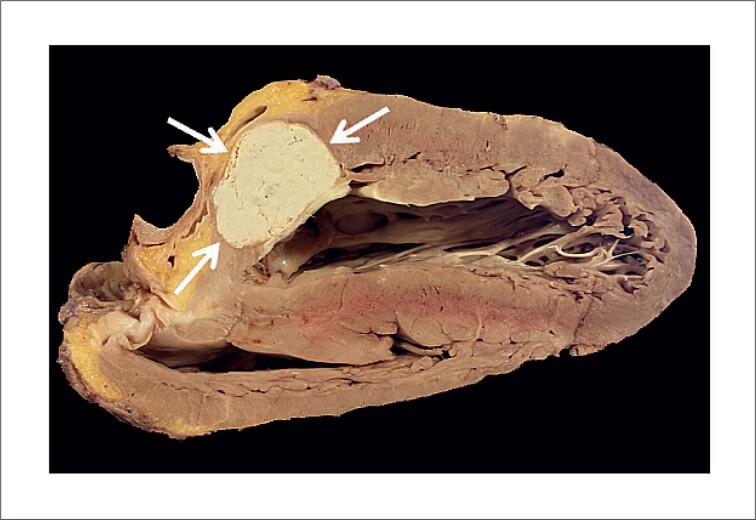
Visão macroscópica da lesão. Coração explantado com lesão de borda parcialmente regular medindo 3,3 x 2,3 cm (setas brancas). O conteúdo da lesão tem aspecto pastoso e semelhante a giz, e foi minimamente descolado durante a secção do espécime cardíaco. É circundado por uma cápsula fibrótica sem continuidade com a cavidade ventricular.

**Figura 4:**
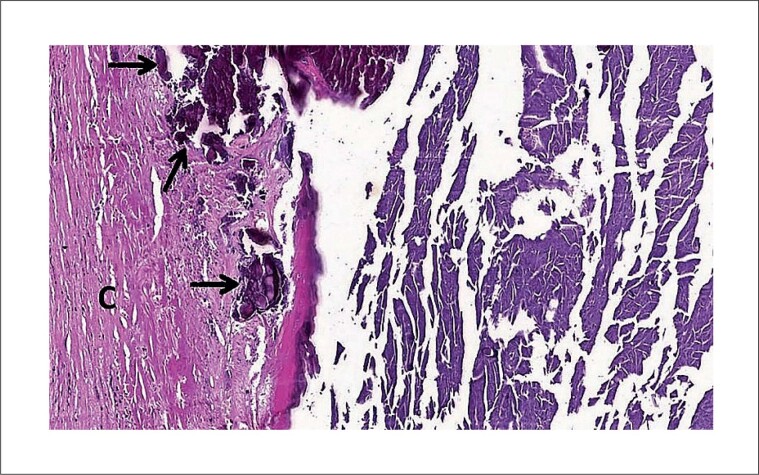
Visão microscópica da lesão – Hematoxilina & eosina, aumento de 200x. O tecido fibrótico da cápsula (C) é mostrado à esquerda, com depósitos de cálcio (setas pretas). Amplo material amorfo e basofílico é visto à direita.

O coração explantado pesava 515 gramas e media 10,5 x 9,5 x 8,0 cm. Todas as quatro câmaras estavam significativamente dilatadas. Uma valva bioprotética estava presente no orifício atrioventricular esquerdo; as outras três valvas eram nativas, incluindo uma valva aórtica mista estenótica e regurgitante, mostrando fusão e encurtamento dos folhetos, altamente sugestivos de cardiopatia reumática. Na porção anterior do miocárdio, na junção atrioventricular esquerda, havia uma massa oval, amarelo-acinzentada, medindo 3,3 x 2,3 cm, com bordas bem definidas e regulares e calcificação periférica fina. Após o coração ter sido seccionado, observou-se o vazamento de uma pequena quantidade de conteúdo pastoso (semelhante ao caseum) (Figura 3). Microscopicamente, a lesão estava envolta por tecido fibrótico com depósitos de cálcio, infiltrado leve de células mononucleares e células gigantes multinucleadas periféricas. No centro da lesão havia abundante material amorfo e basofílico (Figura 4). Todos esses achados são compatíveis com calcificação caseosa do anel mitral (CCAM). No miocárdio do ventrículo esquerdo havia poucos e pequenos agregados de macrófagos e linfócitos, sugestivos de nódulos de Aschoff.

## Discussão

A calcificação do anel mitral (CAM) é uma lesão crônico-degenerativa que acomete principalmente indivíduos idosos, e principalmente mulheres e pacientes com doença em estágio terminal ou anormalidades no metabolismo do cálcio.^1,2^ Geralmente assintomática, a CAM é comumente identificada através do ecocardiograma. A calcificação caseosa do anel mitral (CCAM) é uma variante rara da CAM, representada por uma massa intramiocárdica redonda contendo abundante material pastoso ou espesso, constituído por ácidos graxos, colesterol e cálcio;^1,4,5^ raramente a lesão parece surgir do folheto da valva mitral.^5^ Também geralmente assintomática e mais prevalente em idosos, pode-se suspeitar de CCAM pelo ecocardiograma, que mostra uma massa com bordas distintas e área ecolucente central, sugestiva de liquefação na região do anel mitral.^4,5^ No presente caso, não houve suspeita clínica de CCAM.

Nos exames de ecocardiografia, a CCAM é vista em 0,04% a 0,07% da população geral e em 0,06% dos pacientes com CAM.^5–7^ Sua etiopatogenia é amplamente desconhecida. Consideramos a doença reumática concomitante em nosso paciente como mera coincidência.

Na maioria dos pacientes, a lesão é clinicamente assintomática. Quando presentes, os sinais e sintomas mais comuns estão relacionados à insuficiência mitral.^4^ No presente caso, é difícil atribuir as manifestações clínicas à CCAM, pois muitas delas poderiam ser claramente explicadas pela insuficiência cardíaca causada pela doença reumática e fibrilação atrial. Nos pacientes assintomáticos, um ecocardiograma incidental pode levantar a suspeita. Em nosso paciente, dois ecocardiogramas não foram capazes de detectar a lesão. Em alguns pacientes, o diagnóstico é feito na autópsia^4^ ou em um coração explantado, como observado neste caso.

O diagnóstico de calcificação caseosa do anel mitral é estabelecido pelo exame anatomopatológico. Macroscopicamente, a CCAM é uma lesão arredondada com bordas distintas e uma área central contendo material caseoso, variando em tamanho de 1,5 a 4,0 cm.^8^ Em geral, é encontrado ao redor do anel mitral. Histologicamente,a lesão contém uma camada fibrosa de tecido conjuntivo com depósitos de cálcio, células mononucleares inflamatórias e células gigantes multinucleadas circundando um material amorfo abundante.

Os diagnósticos diferenciais mais importantes incluem mixoma cardíaco, geralmente móvel, pedunculado e localizado ao longo do septo interatrial, abscessos e pseudoaneurismas, cujo conteúdo, embora pastoso, carece de depósitos de cálcio e tumor amorfo cardíaco calcificado, uma lesão constituída por tecido fibroso colagenoso denso com nódulos de cálcio, sem constituinte pastoso.^9–13^

A abordagem terapêutica, em geral conservadora, é pautada pelas implicações clínicas. Em pacientes com insuficiência mitral, a cirurgia cardíaca é indicada.^5^ Além de ser uma intervenção curativa, o procedimento cirúrgico é o melhor método de obtenção de amostras para o diagnóstico morfológico. Às vezes, a lesão regride espontaneamente.^4^ O prognóstico, geralmente bom, depende do tamanho da lesão, sua localização e padrão de crescimento.
