# Supplementary material for: Calcificação Caseosa do Anel Mitral: Diagnóstico Pós-Transplante Cardíaco
Source: Arq Bras Cardiol. 2022 Nov 23;119(6):999–1001. [Article in Portuguese] doi: 10.36660/abc.20210906 (PMC9814813; doi:10.36660/abc.20210906)
Supplement: Translation

# Translation: Caseous Calcification of the Mitral Annulus: A Post-Heart Transplant Diagnosis

**Authors:** Bruno Jordão Chaves, Matheus Bitencourt Duarte, Luiz Guilherme Passaglia, Claudio Gelape, Paulo Hernane Rabelo Azevedo, Geraldo Brasileiro

**Affiliations:** 1 Belo Horizonte MG Brazil Universidade Federal de Minas Gerais - Faculdade de Medicina, Belo Horizonte, MG – Brazil

**Keywords:** Mitral Valve Insufficiency, Calcinosis, Heart Neoplasms/surgery, Heart Transplantation, Cardiomyopathies, Diagnostic, Imaging/methods

## Introduction

Caseous calcified mitral annulus is a non-neoplastic cardiac lesion considered to be a variant of mitral annular calcification that should be suspected when heart masses are detected by echocardiogram, chest x-ray or other radiologic studies.^1–3^ The majority of the patients is asymptomatic, but signs and symptoms of mitral regurgitation, systemic embolization and atrioventricular blocks have been described.³ The diagnosis is confirmed by anatomopathologic examination. The prognosis is good. Here, we present a caseous calcification of the mitral annulus case diagnosed after the pathologic analysis of explanted heart.

## Case report

A 35-year-old man with diagnosis of heart failure (stage III by New York Heart Association Classification) secondary to rheumatic heart disease was admitted at a tertiary public University Hospital for heart transplantation. He had been having recurrent hospitalizations due to chronic heart failure. Physical examination showed edema of the lower limbs, lateral dislocation of apex heartbeat, irregular heart rhythm, presence of the third heart sound, and early systolic and mid-diastolic murmur at the apex of the heart. The chest x-ray revealed a hyperdense lesion localized at the heart next to the mitral valve (Figure 1 and 2). Transthoracic echocardiogram done 3 years before heart transplantation disclosed both right and left atria enlarged, mitral byoprothesis unremarkable and aortic valve regurgitation. Transthoracic echocardiogram performed 2 months before transplantation showed enlargement of both atria and ventricles, moderate bioprosthetic mitral valve insufficiency and moderate aortic valve insufficiency and stenosis (data not shown). In both echocardiograms no calcified lesion was seen around mitral valve annulus.

**Figure 1:** Chest radiography – posteroanterior view. Enlarged heart. Next to the mitral valve, there is an ovulated hyperdense lesion (red arrows) measuring about 3,5cm x 2,0cm. The double density sign (blue arrow) and elevation of the left bronchus (yellow arrow) indicate left atrial enlargement. The left costodiaphragmatic recess is obliterated (black arrow), and a calcified line is noted at the left hemithorax seemingly along the pleura (white arrow).

**Figure 2:** Chest radiography – lateral view. The hyperdense heart lesion (red arrows) and the calcified pleural line (white arrows) are visible.

At the age of 15, the patient went through a bioprosthetic mitral valve replacement to treat the rheumatic mitral valve disease, requiring two subsequent reoperations (age 21 and 23). For the last three years, despite optimal medical therapy, his heart function had continually deteriorated. There were no other comorbidities in his past medical history.

Laboratory findings showed a reduced kidney function (serum creatinine of 3,0 mg/dL and estimated glomerular filtration rate of 25.7 mL/min/1.73m²). Electrocardiogram was remarkable for atrial fibrillation.

The main diagnostic hypothesis was advanced heart failure caused by bioprosthetic mitral valve dysfunction. For a heart mass visualized on the x-ray, some possibilities were considered: mitral annular calcification, calcified myxoma, other cardiac neoplasms or pseudoneoplasms, cardiac abscess, tuberculosis, calcified amorphous tumor or calcified valvar vegetations. The precise diagnosis is obviously essential for the appropriate treatment.

### Management and diagnosis

Given the progressive cardiac failure, the patient went through heart transplantation. During the patient's first postoperative days, severe hemodynamic instability and acute heart failure suggested primary graft rejection. Despite pharmacological intervention for primary graft dysfunction, the patient died after three days of heart transplantation.

The explanted heart weighed 515 grams and measured 10.5 x 9.5 x 8.0 cm. All for chambers were significantly dilated. A bioprosthetic valve was present on the left atrioventricular orifice; the other three valves were native, including a mixed stenotic and regurgitant aortic valve showing fusion and shortening of the leaflets, highly suggestive of rheumatic heart disease. On the anterior portion of the myocardium, at the left atrioventricular junction, there was a gray-yellow, oval mass of 3.3 x 2.3 cm, with well-demarcated and regular borders and thin peripheric calcification. After cutting, a small amount of pasty content (similar to caseum) leaked (Figure 3). Microscopically, the lesion was involved by a fibrotic tissue with calcium deposits, mild mononuclear cell infiltration, and peripheral multinucleated giant cells. In the center of the lesion, there was abundant amorphous and basophilic material (Figure 4). All these findings are consistent with caseous calcification of the mitral annulus (CCMA). In the left ventricle myocardium, there were a few and small macrophage and lymphocyte aggregates suggestive of Aschoff nodules.

**Figure 3:** Macroscopic view of the lesion. Explanted heart with a partially regular border lesion of 3,3 x 2,3 cm (white arrows). The lesion content has a pasty and chalky aspect and was minimally detached during the cutting of the specimen heart. It is involved by a fibrotic capsule without continuity with the ventricle cavity.

**Figure 4:** Microscopic view of the lesion – hematoxylin and eosin, magnification of 200x. On the left, there is the fibrotic tissue of the capsule (C) with calcium deposits (black arrows). A wide amorphous and basophilic material is seen on the right half.

## Discussion

Calcification of mitral annulus (CMA) is a chronic degenerative lesion that affects mainly older people, especially women and patients with end-stage disease or abnormalities in calcium metabolism.^1,2^ Usually asymptomatic, CMA is commonly recognized by echocardiography. Caseous calcification of mitral annulus (CCMA) is a rarely CMA variant represented by a round intramyocardial mass containing abundant pasty or putty-like material composed by fatty acids, cholesterol and calcium;^1,4,5^ rarely, the lesion appears to arise from de mitral valve leaflet.^5^ Also generally asymptomatic and more prevalent in the elderly, CCMA can be suspected by echocardiography, which shows a mass with distinct borders and a central echolucent area suggestive of liquefaction in the annular mitral region.^4,5^ In the current case, CCMA was not clinically suspected.

In echocardiography examinations, CCMA is seen in 0.04% to 0.07% of the general population and in 0.06% of CMA patients.^5–7^ Its ethiopatogenesis is largely unknown. We consider the concomitant rheumatic disease in our patient as merely coincidental.

In most patients, the lesion is clinically asymptomatic. When present, the most common signs and symptoms relate to mitral insufficiency.^4^ In the current case, it is difficult to attribute the clinical manifestations to the CCMA, since many of them could be clearly explained by heart failure due to rheumatic disease and atrial fibrillation. In the asymptomatic patients, an incidental echocardiogram may arise the suspicion. In our patient, two echocardiograms were not able to detect the lesion. In some patients, the diagnosis is made at autopsy^4^ or in an explanted heart, as occurred in this case.

Caseous calcification of mitral annulus’ diagnosis is established by anatomopathological examination. Macroscopically, CCMA is a round lesion with distinct borders and a central area containing caseous material, ranging from 1.5 to 4.0 cm.^8^ In general, it is found around mitral annulus. Histologically, the lesion contains a fibrous layer of connective tissue with calcium deposits, inflammatory mononuclear cells and multinucleated giant cells surrounding an abundant amorphous material.

The most important differential diagnoses include cardiac myxoma, which is usually mobile, pedunculated and located along interatrial septum, abscess and pseudoaneurysms, whose content, although pasty, lacks calcium deposits, and cardiac calcified amorphous tumor, a lesion composed of dense collagenous fibrous tissue with calcium nodules, without pasty constituent.^9–13^

The therapeutic approach, in general conservative, is guided by the clinical repercussions. In patients with mitral regurgitation, cardiac surgery is indicated.^5^ Besides being a curative intervention, surgery is the method to obtain sample for morphological diagnosis. Sometimes, the lesion regress spontaneously.^4^ The prognosis, usually good, depends on its size, location and growth pattern.
